# Treadmill exercise reduces spinal cord injury-induced apoptosis by activating the PI3K/Akt pathway in rats

**DOI:** 10.3892/etm.2013.1451

**Published:** 2013-12-17

**Authors:** SUN-YOUNG JUNG, DAE-YOUNG KIM, TAE YOUNG YUNE, DONG-HOON SHIN, SANG-BIN BAEK, CHANG-JU KIM

**Affiliations:** 1 Department of Physiology, College of Medicine, Kyung Hee University, Seoul 136-701, Republic of Korea; 2 Department of Biochemistry and Molecular Biology, Age-Related and Brain Diseases Research Centre, College of Medicine, Kyung Hee University, Seoul 136-701, Republic of Korea; 3 Department of Food and Biotechnology, Graduate School of Life Sciences and Biotechnology, Korea University, Seoul 136-701, Republic of Korea; 4 Department of Psychiatry, Gangneung Asan Hospital, Ulsan University, Gangneung 210-711, Republic of Korea

**Keywords:** spinal cord injury, treadmill exercise, motor function, apoptosis, neurotrophic factors

## Abstract

Apoptosis occurring secondary to spinal cord injury (SCI) causes further neural damage and functional loss. In this study, a rat model was used to investigate the effect of treadmill exercise on SCI-induced apoptosis and expression of neurotrophic factors. To produce SCI, a contusion injury (10 g × 25 mm) was applied subsequent to laminectomy at the T9–T10 level. Following SCI, treadmill exercise was performed for six weeks. Hindlimb motor function was evaluated with a grid-walking test. The expression of neurotrophic factors and the level of apoptosis at the site of SCI were determined by western blotting. SCI reduced hindlimb motor function and suppressed expression of neurotrophin (NT)-3 and insulin-like growth factor (IGF)-1. Expression of phosphatidylinositol 3-kinase (PI3K), the ratio of phosphorylated Akt to Akt (pAkt/Akt) and the ratio of B-cell lymphoma 2 (Bcl-2) to Bax (Bcl-2/Bax) were decreased, and cleaved caspase-3 expression was increased by SCI. Treadmill exercise enhanced hindlimb motor function and increased expression of nerve growth factor (NGF), NT-3 and IGF-1 in the SCI rats. Treadmill exercise increased PI3K expression, the pAkt/Akt and the Bcl-2/Bax ratios, and suppressed cleaved caspase-3 expression in the injured spinal cord. This study demonstrated that treadmill exercise promotes the recovery of motor function by suppressing apoptosis in the injured spinal cord. The beneficial effect of exercise may be attributed to the increase in expression of neurotrophic factors via activation of the PI3K/Akt pathway.

## Introduction

Spinal cord injury (SCI) is a serious trauma causing severe and often permanent disability. SCI induces primary mechanical damage and causes secondary damage to the spinal cord. Primary damage occurs by mechanical tissue disruption immediately subsequent to trauma. Secondary damage is mediated by complex cellular and molecular processes (1).

Apoptosis, or programmed cell death, is a highly regulated process that enables the elimination of unwanted or dysfunctional cells. However, inappropriate or excessive apoptosis is a component of numerous neurodegenerative conditions, including traumatic injury (2–5). Crowe *et al* (6) observed neuronal apoptosis along the longitudinal axis of the injured spinal cord, which induced deterioration of sensorimotor function. Contusion injury causes apoptosis of neurons, astrocytes, oligodendroglia and microglia in the rat spinal cord (7,8). Apoptosis in SCI is accompanied by activation of caspase-3, a member of the cysteine protease family (9). Apoptosis is regulated by pro- and anti-apoptotic members of the B-cell lymphoma 2 (Bcl-2) protein family (10). Neuronal apoptosis occurs following traumatic brain injury, SCI, seizure and stroke (3,11,12). Therefore, an understanding of apoptotic mechanisms is important for the prevention and treatment of various diseases (9,13,14).

The phosphatidylinositol 3-kinase (PI3K) signaling pathway is implicated in cell survival and apoptosis (15). Akt, also known as protein kinase B, is a main effector in the PI3K signaling pathway and an increase in Akt activity blocks the mitochondrial apoptotic pathway (16,17). The PI3K/Akt signaling pathway mediates mitogen-dependent growth and survival, and inhibition of this pathway results in cell growth arrest and apoptosis (18). Phosphorylation of Akt inactivates the pro-apoptotic factors Bad and procaspase-9, and inhibits apoptosis (19). Inhibition of the phosphorylation of Akt induces apoptosis (20).

A number of neurotrophic factors are implicated in the survival, differentiation and function of central nervous system neurons. Among these factors, brain-derived neurotrophic factor (BDNF), nerve growth factor (NGF), neurotrophin (NT)-3 and insulin-like growth factor (IGF)-1 mediate neuroprotective effects against apoptosis (21,22). Sustained local expression of neurotrophic factors in the sensorimotor cortex and the spinal cord increased axonal sprouting following SCI, providing a basis for the development of neurotrophic factor therapy (23). Acceleration of repair following neuronal injury is accompanied by the upregulation and release of endogenous neurotrophic factors (24). Exogenous NGF is critical in neuronal plasticity, regeneration and prevention of apoptosis following traumatic brain injury (25).

Exercise exerts neuroprotective effects by enhancing neurogenesis, increasing the expression of neurotrophic factors and inhibiting apoptosis (3,26,27). Griesbach *et al* (28) reported that voluntary wheel exercise improved functional recovery from traumatic brain injury via an endogenous mechanism involving upregulation of BDNF and IGF-1 expression. Treadmill exercise may improve functional recovery from SCI (29,30). However, to the best of our knowledge, detailed mechanisms of the impact of treadmill exercise on SCI have not been described. In this study, a rat model was used to investigate the effect of treadmill exercise on SCI-induced apoptosis and expression of neutrotrophic factors.

## Materials and methods

### Animals and treatments

Male Sprague Dawley rats (180±10 g, 6 weeks old, n=32) were purchased the Daehan Biolink Co. (Chungbuk, Korea), and individually housed in plastic cages with a controlled temperature (20±2°C) and a 12-h light/dark cycle (lights on from 7:00 a.m. to 7:00 p.m.). Food and water were available *ad libitum*. The rats were divided into four groups: Sham surgery, sham surgery plus exercise, SCI and SCI plus exercise (n=8/group). This study was performed in accordance with the guidelines of the National Institutes of Health and the Korean Academy of Medical Sciences (Seoul, Korea), and approved by Kyung Hee University Institutional Animal Care and Use Committee (Seoul, Korea).

### Surgical procedures

The surgical procedure for inducing SCI was conducted according to established methods (31). The rats were anesthetized with chloral hydrate (500 mg/kg, intraperitoneal), and a laminectomy was performed at the T9–T10 level, exposing the underlying cord without disrupting the dura. The spinous processes of the T8–T11 level were clamped to stabilize the spine, and the exposed dorsal surface of the cord was subjected to contusion injury using the New York University impactor (New York University, New York, NY, USA) as previously described (31). A moderate contusion was created by dropping a 10-g rod (2.5 mm in diameter) from a height of 12.5 mm onto the exposed cord. Following SCI, urinary bladders were emptied twice a day for one week and thereafter when necessary. Rats in the sham surgery and the sham surgery plus exercise groups received a T10 laminectomy without weight-drop contusion injury.

### Treadmill exercise

At one week post-surgery, the rats in the exercise groups were trained to walk on the treadmill. When no stepping of the hindlimb occurred in response to the moving treadmill and stepping of the forelimb, movement was elicited by manual stimulation of the perineum. The exercise sessions consisted of three minutes four times per day during the first week, and five minutes six times per day from the second to the sixth week at a speed of 6 m/min, with five minutes resting time between each session. Treadmill exercise was performed six days per week.

### Grid-walking test

The grid-walking test was conducted as previously described (32). This method measures the ability of animals to control hindlimb placing and is regarded as an indicator of corticospinal function. Prior to surgery, the rats were trained to walk on a runway of metal grid bars elevated from the ground. A grid pathway measuring 1.2 m in length was used, and the rats were allowed to walk three times voluntarily across the pathway. The number of errors (hindlimb stepping through the grid) was counted and the three values were averaged to produce a single error value per rat.

### Western blot analysis

Western blotting was performed following standard methods (2,3). Spinal cord tissue was collected and immediately frozen at −70°C. The tissues were homogenized with lysis buffer [50 mM Tris-HCl at pH 8.0, 150 mM NaCl, 10% glycerol, 1% Triton X-100, 1.5 mM MgCl_2_·6H_2_O, 1 mM ethylene glycol tetraacetic acid (EGTA), 1 mM phenylmethylsufonyl fluoride (PMSF), 1 mM Na_2_VO_4_ and 100 mM NaF] and centrifuged at 900 × g for 30 min. Protein content was measured using a colorimetric protein assay kit (Bio-Rad, Hercules, CA, USA). Equal amounts of protein (30 μg) were loaded onto 12% polyacrylamide gels and separated by SDS-PAGE. Separated proteins were transferred from the gels to a nitrocellulose membrane. To evaluate the expression of neurotrophic factors, rabbit polyclonal anti-NT-3 antibody (1:1,000; Santa Cruz Biotechnology Inc., Santa Cruz, CA, USA), goat polyclonal anti-IGF-1 antibody (1:1,000; Santa Cruz Biotechnology Inc.) and rabbit polyclonal anti-NGF antibody (1:1,000; Santa Cruz Biotechnology Inc.) were used as the primary antibodies. In addition, mouse monoclonal anti-PI3K antibody (1:1,000; Santa Cruz Biotechnology, Inc.), rabbit polyclonal anti-phosphorylated (p)Akt and anti-Akt antibodies (1:1,000; Cell Signaling Technology, Inc., Beverly, MA, USA), mouse monoclonal anti-Bcl-2 and Bax antibodies (1:1,000; Santa Cruz Biotechnology, Inc.) and rabbit polyclonal anti-cleaved caspase-3 antibody (1:1,000; Cell Signaling Technology, Inc.) were used as the primary antibodies. Horseradish peroxidase-conjugated anti-mouse antibody for PI3K, Bcl-2 and Bax (1:3,000; Vector Laboratories, Burlingame, CA, USA), horseradish peroxidase-conjugated anti-rabbit antibody for NT-3, NGF, pAkt, Akt and cleaved caspase-3 (1:5,000; Vector Laboratories), and horseradish peroxidase-conjugated anti-goat antibody for IGF-1 (1:5,000; Vector Laboratories) were used as the secondary antibodies. Bands were visualized using an enhanced chemiluminescence (ECL) kit (Santa Cruz Biotechnology Inc.). To compare the expression level of proteins, bands were calculated densitometrically using Image-Pro^®^ Plus software (Media Cybernetics, Silver Spring, MD, USA).

### Statistical analysis

All data were analyzed using SPSS 20.0 statistical software (SPSS Inc., Chicago, IL, USA). The data are expressed as the mean ± standard error of the mean (SEM). For comparisons among the groups, one-way analysis of variance (ANOVA) and Duncan’s post hoc test were performed. P<0.05 was considered to indicate a statistically significant difference.

## Results

### Treadmill exercise improves hindlimb motor function following SCI

**Figure 1:**
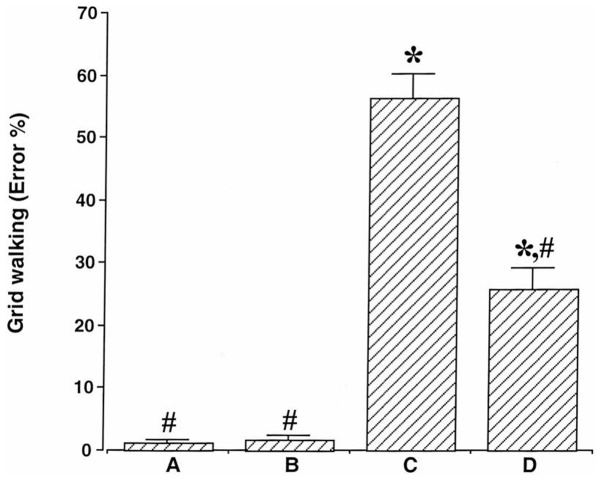
Effect of treadmill exercise on recovery of motor function. (A) Sham surgery group; (B) sham surgery plus exercise group; (C) spinal cord injury (SCI) group; (D) SCI plus exercise group. Data are presented as the mean ± standard error of the mean. ^*^P<0.05 compared with the sham surgery group, ^#^P<0.05 compared with the SCI group.

The rats’ performance was monitored by counting the number of mis-steps and accurate footsteps. Prior to induction of SCI, all rats had highly accurate foot placement when walking on the grid. Following SCI, the error ratio was 1.10±0.70% in the sham surgery group, 1.65±0.64% in the sham surgery plus exercise group, 56.30±3.86% in the SCI group and 25.63±3.52% in the SCI plus exercise group (Fig. 1). The error ratio increased following SCI (P<0.05). By contrast, treadmill exercise reduced the error ratio (P<0.05), showing that treadmill exercise improved motor function in the hindlimb following SCI.

### Treadmill exercise enhances the expression of neurotrophic factors following SCI

**Figure 2:**
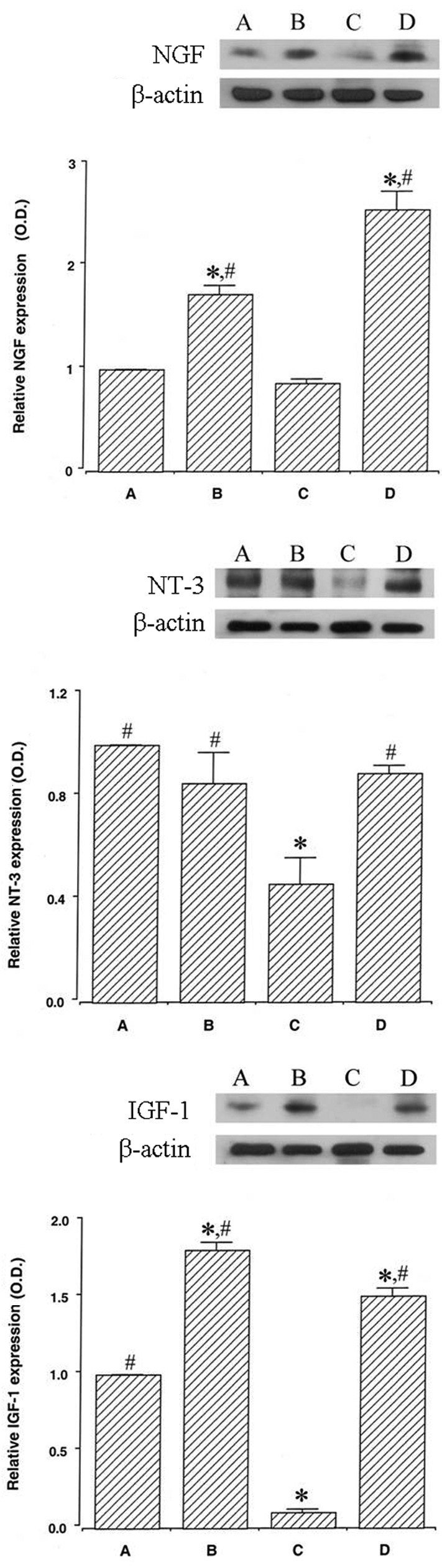
Effect of treadmill exercise on the expression of neurotrophic factors (NGF, NT-3 and IGF-1) in the spinal cord. (A) Sham surgery group; (B) sham surgery plus exercise group; (C) spinal cord injury (SCI) group; (D) SCI plus exercise group. Data are presented as the mean ± standard error of the mean. ^*^P<0.05 compared with the sham surgery group; ^#^P<0.05 compared with the SCI group. NGF, nerve growth factor; NT-3, neurotrophin-3; IGF-1, insulin-like growth factor-1; O.D., optical density.

Protein expression of NGF (13 kDa) was set at 1.00 in the sham surgery group, with expression levels of 1.73±0.08 in the sham surgery plus exercise group, 0.86±0.04 in the SCI group and 2.55±0.18 in the SCI plus exercise group (Fig. 2, upper panel). Expression of NGF was not changed by SCI, while treadmill exercise enhanced NGF expression in sham-operated and SCI rats (P<0.05).

Setting the expression of NT-3 (21 kDa) in the sham surgery group at 1.00, the expression of NT-3 was 0.85±0.12 in the sham surgery plus exercise group, 0.46±0.10 in the SCI group and 0.89±0.03 in the SCI plus exercise group (Fig. 2, middle panel). The expression of NT-3 was reduced following SCI (P<0.05); however, treadmill exercise enhanced NT-3 expression in the SCI rats (P<0.05).

Setting the expression of IGF-1 (17 kDa) in the sham surgery group at 1.00, the expression of IGF-1 was 1.81±0.05 in the sham surgery plus exercise group, 0.10±0.02 in the SCI group and 1.51±0.05 in the SCI plus exercise group (Fig. 2, lower panel). The expression of IGF-1 was reduced following SCI (P<0.05); however, treadmill exercise was observed to enhance IGF-1 expression in sham-operated and SCI rats (P<0.05).

### Treadmill exercise enhances the expression of PI3K following SCI

**Figure 3:**
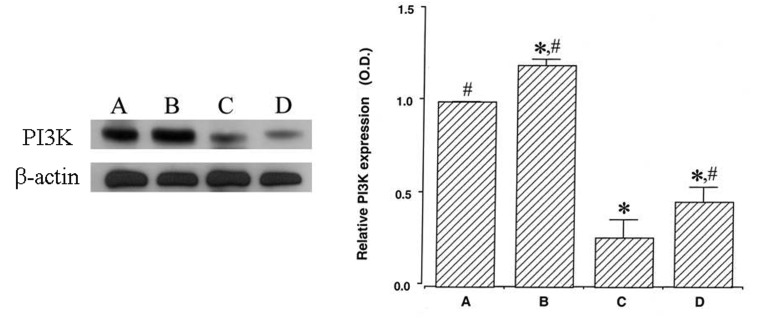
Effect of treadmill exercise on the expression of phosphatidylinositol 3-kinase (PI3K) in the spinal cord. (A) Sham surgery group; (B) sham surgery plus exercise group; (C) spinal cord injury (SCI) group; (D) SCI plus exercise group. Data are presented as the means ± standard error of the mean. ^*^P<0.05 compared with the sham surgery group, ^#^P<0.05 compared with the SCI group.

When the expression of PI3K (85 kDa) in the sham surgery group was set at 1.00, the expression of PI3K was 1.20±0.03 in the sham surgery plus exercise group, 0.27±0.09 in the SCI group and 0.46±0.08 in the SCI plus exercise group (Fig. 3). Expression of PI3K was reduced following SCI (P<0.05); however, treadmill exercise enhanced PI3K expression in sham-operated and SCI rats (P<0.05).

### Treadmill exercise enhances the ratio of pAkt to Akt following SCI

**Figure 4:**
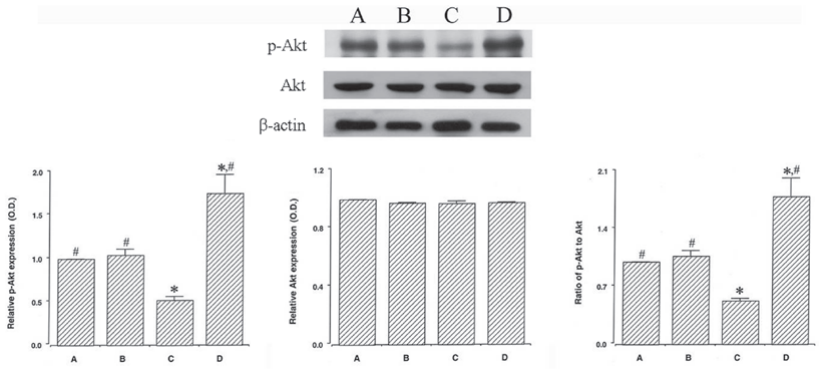
Effect of treadmill exercise on the ratio of phosphorylated (p)Akt/Akt in the spinal cord. (A) Sham surgery group; (B) sham surgery plus exercise group, (C) spinal cord injury (SCI) group; (D) SCI plus exercise group. Data are presented as the mean ± standard error of the mean. ^*^P<0.05 compared with the sham surgery group, ^#^P<0.05 compared with the SCI group.

When the expression of pAkt (60 kDa) in the sham surgery group was set at 1.00, the expression of pAkt was 1.04±0.07 in the sham surgery plus exercise group, 0.52±0.04 in the SCI group and 1.75±0.22 in the SCI plus exercise group (Fig. 4, left panel). Expression of pAkt was reduced following SCI (P<0.05). Treadmill exercise enhanced pAkt expression in the SCI rats (P<0.05).

When the expression of Akt (60 kDa) in the sham surgery group was set at 1.00, the expression of Akt was 0.97±0.01 in the sham surgery plus exercise group, 0.96±0.02 in the SCI group and 0.97±0.01 in the SCI plus exercise group (Fig. 4, middle panel). Expression of Akt was not changed by induction of SCI, and treadmill exercise exerted no significant effect on Akt expression in either sham-operated or SCI rats.

When the ratio of pAkt/Akt in the sham surgery group was set at 1.00, the ratio of pAkt/Akt was 1.07±0.07 in the sham surgery plus exercise group, 0.53±0.03 in the SCI group and 1.79±0.23 in the SCI plus exercise group (Fig. 4, right panel). The pAkt/Akt ratio was reduced following SCI (P<0.05). Treadmill exercise enhanced the pAkt/Akt ratio in SCI rats (P<0.05).

### Treadmill exercise enhances the ratio of Bcl-2 to Bax following SCI

**Figure 5:**
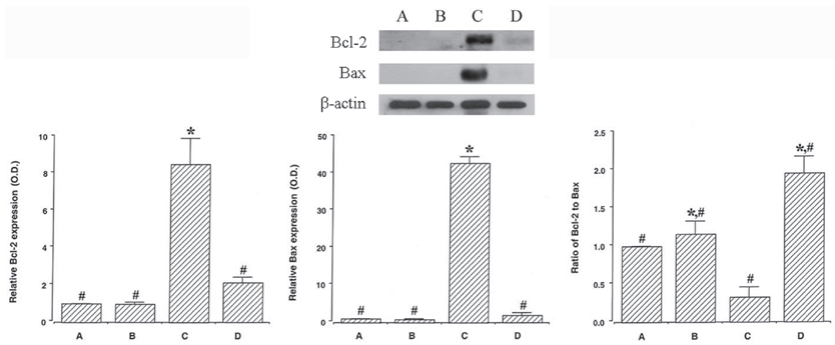
Effect of treadmill exercise on the ratio of Bcl 2/Bax in the spinal cord. (A) Sham surgery group; (B) sham surgery plus exercise group; (C) spinal cord injury (SCI) group; (D) SCI plus exercise group. Data are presented as the mean ± standard error of the mean. ^*^P<0.05 compared with the sham surgery group, ^#^P<0.05 compared with the SCI group.

When the expression of Bcl-2 (28 kDa) in the sham surgery group was set at 1.00, the expression of Bcl-2 was 0.99±0.09 in the sham surgery plus exercise group, 8.52±3.43 in the SCI group and 2.12±0.74 in the SCI plus exercise group (Fig. 5, left panel). Expression of Bcl-2 increased following SCI (P<0.05), while treadmill exercise suppressed Bcl-2 expression in the SCI rats (P<0.05).

When the expression of Bax (23 kDa) in the sham surgery group was set at 1.00, the expression of Bax was 0.90±0.12 in the sham surgery plus exercise group, 42.88±3.31 in the SCI group and 2.02±0.69 in the SCI plus exercise group (Fig. 5, middle panel). Expression of Bax increased following SCI (P<0.05), while treadmill exercise suppressed Bax expression in the SCI rats (P<0.05).

When the ratio of Bcl-2/Bax in the sham surgery group was set at 1.00, the ratio of Bcl-2/Bax was 1.16±0.17 in the sham surgery plus exercise group, 0.33±0.13 in the SCI group and 1.96±0.63 in the SCI plus exercise group (Fig. 5, right panel). The ratio of Bcl-2/Bax was decreased by SCI (P<0.05) and treadmill exercise enhanced the Bcl-2/Bax ratio in sham-operated and SCI rats (P<0.05).

### Treadmill exercise suppresses the expression of cleaved caspase-3 following SCI

**Figure 6:**
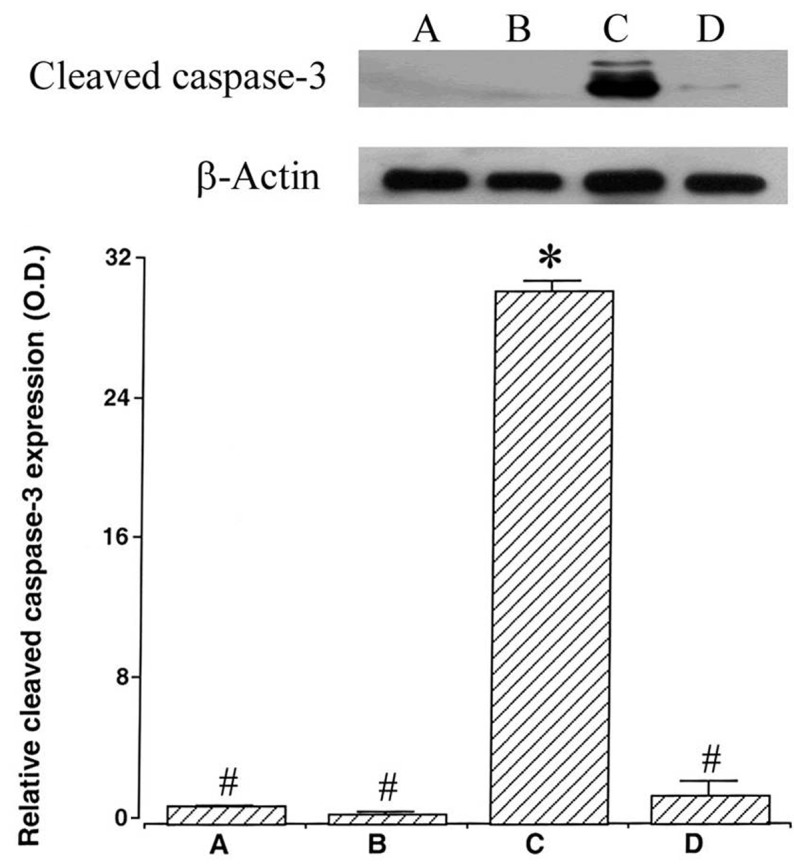
Effect of treadmill exercise on the expression of caspase-3 in the spinal cord. (A) Sham surgery group; (B) sham surgery plus exercise group; (C) spinal cord injury (SCI) group; (D) SCI plus exercise group. Data are presented as the mean ± standard error of the mean. ^*^P<0.05 compared with the sham surgery group, ^#^P<0.05 compared with the SCI group.

When the expression of cleaved caspase-3 (17 kDa, 19 kDa) in the sham surgery group was set at 1.00, the expression of cleaved caspase-3 protein was 0.63±0.10 in the sham surgery plus exercise group, 30.38±12.61 in the SCI group and 1.65±0.78 in the SCI plus exercise group (Fig. 6). The expression of cleaved caspase-3 was increased by SCI (P<0.05), while treadmill exercise suppressed cleaved caspase-3 expression in the SCI rats (P<0.05).

## Discussion

The exploration of different models of SCI has suggested that physical exercise may facilitate functional recovery (29,30). A study by de Leon and Acosta (33) suggested that robot-assisted training generates hindlimb sensory stimuli that effectively enhance the ability of the lumbar spinal cord to produce hindlimb stepping. Treadmill exercise improved the use of a paretic hindlimb, decreased muscle atrophy and increased axonal regrowth and collateral sprouting proximal to the lesion site in thoracic hemisectioned mice (29). In the present study, treadmill exercise enhanced hindlimb motor function in the SCI rats as assessed by grid-walking, showing that treadmill exercise improved hindlimb motor function following SCI.

Neurotrophic factors, including NGF, NT-3 and IGF-1, modulate neuronal growth, differentiation and survival (34). Neurotrophic factors protect against various brain injuries, and these therapeutic effects are mediated by the PI3K/Akt signaling pathway (35,36). Ying *et al* (37) reported that voluntary wheel running increased expression of NT-3 and its tyrosine kinase C receptor in the spinal cord. Statins improved neurological outcomes in an experimental intracerebral hemorrhage model through activation of the PI3K/Akt signaling pathway (35). The anti-apoptotic effect of quercetin on focal cerebral ischemia may be associated with activation of the PI3K/Akt signaling pathway (36). In the present study, expression of NT-3 and IGF-1 was reduced in the spinal cord following SCI. NGF levels in the SCI rats were not significantly different from those in the sham-operated rats. By contrast, treadmill exercise increased expression of NGF, NT-3 and IGF-1 expression in SCI rats. These results suggested that treadmill exercise exerted a protective effect on SCI by stimulating the expression of neurotrophic factors.

Intracellular pro-apoptotic signals induce mitochondrial outer membrane permeabilization followed by release of cytochrome *c* into the cytosol (38). As a result, caspase-9 is activated and induces the activation of caspase-3 (39). The PI3K/Akt pathway is involved in apoptosis regulation (15). The PI3K/Akt pathway is implicated in anti-apoptotic mechanisms, and this pathway influences the balance of anti- and pro-apoptotic proteins such as Bcl-2 and Bax, respectively (40). An *ex vivo* study using human monocytes demonstrated that activation of PI3K/Akt signaling suppressed apoptosis (40). By contrast, in a human prostate cancer cell line, inhibition of the PI3K/Akt signaling pathway exerted a pro-apoptotic effect (18). Exercise-induced enhancement of neuronal survival is associated with the increased expression of several key intermediates of the PI3K/Akt pathway (41). In the present study, PI3K expression and the ratio of pAkt/Akt were reduced in the spinal cord following SCI. However, treadmill exercise increased PI3K levels and enhanced the ratio of pAkt/Akt in the injured spinal cord. These results suggested that exercise-induced activation of the PI3K/Akt signaling pathway may be correlated with the anti-apoptotic effect of treadmill exercise following SCI.

The Bcl-2 family includes anti-apoptotic molecules, including Bcl-2, and pro-apoptotic molecules, including Bax, Bid and Bad. The Bcl-2 family is critical in determining whether neurons survive or die (42). Cycling exercise increased Bcl-2 mRNA expression, and high levels of Bcl-2 mRNA expression correlated with reduced expression of caspase-7 and caspase-9 mRNAs in SCI rats (43). Treadmill exercise suppressed Bax expression and increased Bcl-2 expression in the hippocampus following traumatic brain injury (3). The ratio of Bcl-2/Bax determines the mitochondrial response to apoptotic stimuli and is crucial in determining whether cells survive or undergo apoptosis (2,44). In the present study, the ratio of Bcl-2/Bax in the spinal cord was decreased by SCI, while treadmill exercise increased the ratio of Bcl-2/Bax in the injured spinal cord, by enhancing expression of the anti-apoptotic Bcl-2 protein. Under the experimental conditions of the present study, SCI increased cleaved caspase-3 expression in the injured spinal cord. By contrast, treadmill exercise suppressed cleaved caspase-3 expression in the SCI rats. These results suggested that treadmill exercise exerted an anti-apoptotic effect on the injured spinal cord.

The present study demonstrated that treadmill exercise promotes the recovery of hindlimb motor function through suppressing apoptosis in the injured spinal cord. The anti-apoptotic effect of exercise may result from the exercise-mediated increase in the expression of neurotrophic factors via activation of the PI3K/Akt pathway. Based on these results, treadmill exercise may exert a protective effect and improve the recovery of locomotion following SCI.
